# Relationship between Prenatal or Postnatal Exposure to Pesticides and Obesity: A Systematic Review

**DOI:** 10.3390/ijerph18137170

**Published:** 2021-07-04

**Authors:** Helena Pinos, Beatriz Carrillo, Ana Merchán, Judit Biosca-Brull, Cristian Pérez-Fernández, María Teresa Colomina, Fernando Sánchez-Santed, Fernando Martín-Sánchez, Paloma Collado, Jorge L. Arias, Nélida M. Conejo

**Affiliations:** 1 Department of Psychobiology, Faculty of Psychology, National Distance Education University (UNED), 28040 Madrid, Spain; bcarrillo@psi.uned.es (B.C.); pcollado@psi.uned.es (P.C.); 2 Joint Research Institute-UNED-Instituto de Salud Carlos III (IMIENS), 28029 Madrid, Spain; fmartin@isciii.es; 3 Department of Psychology and Health Research Center (CEINSA), Almeria University, 04120 Almeria, Spain; anamercar@gmail.com (A.M.); cpf603@ual.es (C.P.-F.); fsanchez@ual.es (F.S.-S.); 4 Research in Neurobehavior and Health (NEUROLAB), Universitat Rovira i Virgili, 43007 Tarragona, Spain; judit.biosca@urv.cat (J.B.-B.); mariateresa.colomina@urv.cat (M.T.C.); 5 Department of Psychology and Research Center for Behavior Assessment (CRAMC), Universitat Rovira i Virgili, 43007 Tarragona, Spain; 6 National School of Public Health, Institute of Health Carlos III, University Institute of Research-UNED-Institute of Health Carlos III (IMIENS), 28029 Madrid, Spain; 7 Laboratory of Neuroscience, Department of Psychology, Instituto de Neurociencias del Principado de Asturias (INEUROPA), University of Oviedo, 33003 Oviedo, Spain; jarias@uniovi.es; 8 Instituto de Investigación Sanitaria del Principado de Asturias (ISPA), 33006 Oviedo, Spain

**Keywords:** obesity, pesticides, organophosphate, organochlorine, chlorpyrifos, carbamates, pyrethroids, neonicotinoids

## Abstract

In recent years, the worldwide prevalence of overweight and obesity among adults and children has dramatically increased. The conventional model regarding the onset of obesity is based on an imbalance between energy intake and expenditure. However, other possible environmental factors involved, such as the exposure to chemicals like pesticides, cannot be discarded. These compounds could act as endocrine-disrupting chemicals (EDC) that may interfere with hormone activity related to several mechanisms involved in body weight control. The main objective of this study was to systematically review the data provided in the scientific literature for a possible association between prenatal and postnatal exposure to pesticides and obesity in offspring. A total of 25 human and 9 animal studies were analyzed. The prenatal, perinatal, and postnatal exposure to organophosphate, organochlorine, pyrethroid, neonicotinoid, and carbamate, as well as a combined pesticide exposure was reviewed. This systematic review reveals that the effects of pesticide exposure on body weight are mostly inconclusive, finding conflicting results in both humans and experimental animals. The outcomes reviewed are dependent on many factors, including dosage and route of administration, species, sex, and treatment duration. More research is needed to effectively evaluate the impact of the combined effects of different pesticides on human health.

## 1. Introduction

Obesity has been defined by the World Health Organization (WHO) as a chronic disease of epidemic global proportions (WHO, 2014). Approximately 13% of the world’s current population is obese (WHO. Available at: https://www.who.int/director-general/speeches/detail/who-director-general-s-opening-remarks-at-obesity-setting-the-global-agenda-event-4-march-2021 accessed on 17 May 2021). Obesity appears to be a multifactorial disease with biological, psychosocial, and behavioral factors that include genetic, socioeconomic, and cultural influences [1,2,3]. Traditional approaches to obesity have considered that the imbalance between energy consumed and the energy expended is the foundation of this disease. However, the worldwide rapid increase in overweight and obesity prevalence has shifted the focus onto other possible environmental factors, such as pesticides. These chemicals may contribute to the onset of obesity-related diseases [4]. Therefore, obesity studies are particularly well suited for new research approaches based on the Exposome concept [5].

Pesticides comprise a heterogeneous group of compounds that are released into the environment during agriculture activities to control home pests or for water chlorination [6]. These substances are harmful, and their compounds may enter the food chain, exposing humans to the toxic actions of pesticides. Throughout life, the major source of human exposure to pesticides is due to the consumption of contaminated food [7,8]. However, there is another source of prenatal exposure in children whose mothers are living or working in areas particularly exposed to pesticides due to a greater presence of agricultural or industrial activities. Interestingly, more than one in two adults and nearly one in six children are overweight or obese in the Organization for Economic Co-operation and Development (OECD) area (https://www.oecd-ilibrary.org/sites/7402dbb2-en/index.html?itemId=/content/component/7402dbb2-en accessed on 17 May 2021).

The effects of pesticides on health are not limited to their release time as some pesticides can persist in the environment during months or even years [9]. Special mention should be made to dichlorodiphenyltrichloroethane (DDT) due to a persistence related to its half-life of up to 30 years in the environment [10]. DDT metabolites have the same stability in the environment and show bioaccumulation in the organism, similar to polychlorinated biphenyls (PCBs), and can be bioaccumulated in the food chain and finally in the human body because of their lipophilic nature [9,11].

Studies analyzing the effect of pesticides on health have been carried out mostly on organochlorine pesticides (OCPs) [12,13]. More specifically, organochlorines and organophosphorus are the most widely studied insecticides linked to obesity and/or type 2 diabetes mellitus (T2DM) in humans and rodents [14]. OCPs could act as endocrine-disrupting chemicals (EDC) that may damage the mechanism of weight control [15]. According to the Endocrine Society, an endocrine disruptor can be defined as an exogenous chemical that interferes in any aspect of hormonal activity [16]. EDCs have several unique features that distinguish them from other common chemicals. They also include various lipophilic compounds that accumulate mainly in lipid-containing tissues, like adipose tissue, and move, bound to lipids, within the body [17]. Some animal studies have demonstrated that EDC exposure during development can act on the food intake circuit and lead to weight gain by means of multiple mechanisms [18]. The hypothesis of developmental origins of health and disease by David Baker suggests that there are critical windows during development in which environmental disruptions can lead to subtle changes in multiple biological mechanisms (gene expression, tissue organization) that could lead to permanent dysfunction and increased susceptibility to developing many diseases later in life [19,20]. For example, low birth weight due to maternal malnutrition results in increased susceptibility to obesity, stroke, coronary heart disease, and metabolic syndrome (for review see [20]).

Additionally, it is possible that epigenetic changes in cells or tissues during development play a role in these diseases. In fact, some chronic diseases include obesity and can be linked to epigenetic modifications as a result of early exposure to several environmental factors [18].

In the same way, exposure to pesticides can result in weight gain through different pathways. Pesticides can alter the function of the adipose tissue, increasing adipocyte differentiation and proliferation, as well as lipid uptake by increasing the number or volume of adipocytes [21]. Furthermore, pesticides can alter the neuroendocrine control of feeding and nutrient metabolism, impairing the function of brain regions involved in these functions [21]. In fact, these substances have been called “obesogenic” [16,22] based on their effects on energy metabolism that can lead to obesity and metabolic syndrome [11].

It is worth mentioning that whilst some authors reported an increased risk of T2DM or obesity in populations exposed to pesticides [6,21,23], other authors reported only a limited association [24]. However, it is difficult to conduct studies with experimental animals using different pesticides and to determine the underlying alterations observed in human studies. Furthermore, it seems more difficult to demonstrate a direct effect of pesticides in humans (for review see [25]).

In addition, other types of insecticides such as carbamates, pyrethroids and neonicotinoids have also been associated with the development of obesity and/or T2DM in humans and rodents [26,27,28]. In particular, pyrethroids and neonicotinoids have been involved in potentiated adipogenesis and/or altered glucose responsiveness, as known risk factors for obesity and T2DM, respectively [29].

Considering the great interest, it may have to jointly review data on the effects of pesticides on energy metabolism, the main objective of this study was to perform a systematic review-based strategy to evaluate and integrate the evidence from clinical and preclinical studies in order to assess a possible association between prenatal and postnatal exposure to pesticides and obesity in offspring from human and animal studies.

## 2. Materials and Methods

### 2.1. Review Protocol

Before carrying out the literature search, a detailed review protocol was created in accordance with the “Preferred Reporting Items for Systematic Review and Meta-Analysis Protocols” (PRISMA-P) [30].

### 2.2. Eligibility Criteria

As recommended by PRISMA, we initially organized this systematic review according to the principles stated in the PICOS acronym (Participants, Interventions, Comparators, Outcome measures, Study design).

The PICOS criteria were identified as follows:

− Types of participants: young humans (children or adolescents) and rodents.
− Types of interventions: prenatal, perinatal, or postnatal environmental exposure to pesticides.
− Types of Comparators: studies comparing the prenatal or postnatal environmental exposure to pesticides, with either a control group, a non-exposed group, or between groups with different levels of exposure (i.e., low, medium, high).
− Types of outcome measures: obesity, overweight, or metabolic syndrome, measured through body weight, height, body mass index, and/or waist circumference. Furthermore, biological outcomes such as temperature, organ weight, biochemical measurements, and brain histomorphological alterations were assessed. Additionally, behavioral measures related to cognitive impairment and emotional disturbances were also assessed.
− Types of study design: experimental studies for literature with animal models, and cohort, cross-sectional in the case of human studies. In this way, rodent models have shown the highest face validity to reproduce the human metabolic syndrome induced by high-carbohydrate and high-fat diets [31]. In addition, many current studies have addressed the induction of metabolic syndrome in rodents [32]. Thus, a recent study refers to the main criteria and reference values used to reproduce this disease in animal models [33].

All articles included were published between the years of 2005 and 2021 and were written in English.

Exclusion criteria were those that did not meet the previous defined PICOS characteristics. Therefore, we did not select case studies, reviews, abstracts or communication to scientific meetings, or qualitative studies. In addition, only articles published in peer-review journals were included.

### 2.3. Information Sources

Comprehensive literature searches of Pubmed, Toxnet and Scopus were conducted in April 2021. The filters employed in the database searches were language (English) and date of publication (2005–2021). The search formula was: (“pesticides” OR “herbicides” OR “insecticides”) AND (“obesity” OR “overweight” OR “metabolic syndrome” OR “exercise”) AND (“prenatal” OR “postnatal” OR “long-term effects” OR “child” OR “adolescent” OR “maternal exposure” OR “offspring”). In Pubmed, MeSH terms were employed in the literature search. Furthermore, a manual search was carried out in relevant journals and in the reference lists of reviews focusing on the subject.

### 2.4. Study Selection and Data Collection Process

The complete lists of results from the three database searches were examined for eligibility in an unblinded manner by two reviewers. Relevant decisions were agreed upon with the research team through discussion until consensus.

Data extraction was conducted, in an unblinded manner, independently by two reviewers. Disagreements based on the extracted data were resolved through discussion until consensus was reached.

### 2.5. Risk of Bias in Individual Studies

For human studies, the Newcastle-Ottawa Scale (NOS) [34] was used. The NOS encompasses 3 quality parameters: selection, comparability, and exposure/outcome assessment. It assigns a maximum of 4 points for selection, 2 points for comparability, and 3 points for exposure or outcome (for a total of 9 points). Hence, the total quality index score was ranked as follows: 0 to 2, 3 to 4, 5 to 6, and 7 to 9 which correspond to low (L), medium high (MH), high (H), and very high (VH) quality, respectively.

“SYRCLE’s tool for assessing risk of bias” [35], based on the Cochrane Collaboration RoB Tool [36] and adapted to aspects of bias in animal experiments, was used to assess methodological quality of the included animal studies.

In this regard, the SYRCLE encompasses 5 quality parameters: selection, performance, detection, attrition, and reporting bias. It assigns a maximum of 6 points for selection, 4 points for performance, 4 points for detection, 4 points for attrition and 4 points for reporting (for a total of 18 points). Therefore, the total quality index score ranked as follows: 0 to 3, 4 to 6, 7 to 9, 10 to 12, 13 to 15, and 16 to 18 which correspond to very low (VL), low (L), medium low (ML), medium high (MH), high (H), and very high (VH) quality, respectively.

### 2.6. Summary Measures and Analysis

We were unable to perform a meta-analysis given the heterogeneity observed among the human and animal studies. Therefore, we conducted a descriptive and critical review following the aforementioned protocol. Our summary measures take the form of a qualitative interpretation and a narrative analysis.

## 3. Results

### 3.1. Study Selection

**Figure 1:**
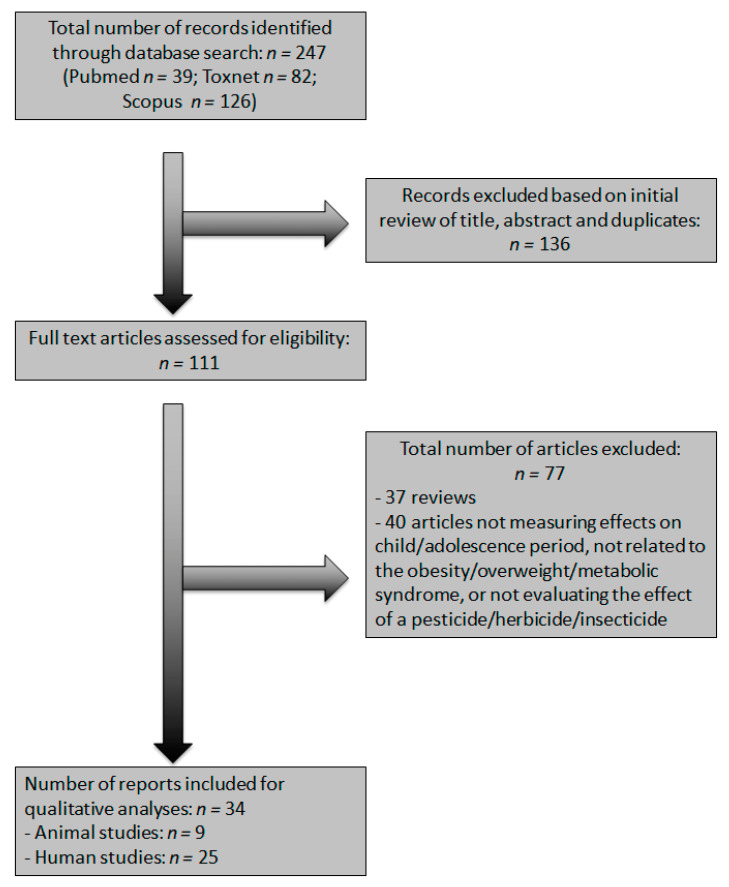
PRISMA (Preferred Reporting Items for Systematic Reviews and Meta-Analyses) flow chart.

The flow chart shows the complete search strategy (Figure 1). The systematic search identified 247 references, of which 116 articles were included for the full-text review. Of the remaining articles, reviews and articles which did not meet the inclusion criteria were removed. Finally, 9 animal studies and 25 human studies (23 cohorts and 2 crossover experimental designs) were included in our systematic review.

### 3.2. Clinical Studies Characteristics

A total of 25 clinical studies were accepted for revision. Most studies were prospective cohort studies labeled with VH quality (22 out 25, 88%) [37,38,39,40,41,42,43,44,45,46,47,48,49,50,51,52,53,54,55,56,57,58], except study [59] which is H quality. Two of the 25 studies conducted cross-sectional studies with VH [60] or H [61] quality scores. Cohorts were from Europe, America, and Asia (see Table 1). A total of 44% of the studies were conducted in children whose mothers had been exposed to pesticides during pregnancy due to their work or living conditions, and the others were carried out with the general population. The area of the population from the studies was rural (36%), urban (52%), or both (12%). Socioeconomic and health variables were considered in all the studies. With regard to the age of the participants included in the studies, five out of the 25 studies (20%) were carried out at birth or within six months and 13 out of 25 (52%) were conducted during childhood up to 9 years of age. A total of six out of 25 (24%) were carried out with teenage participants, in some cases with a follow up period up to 18 years of age [37,60] or up to 22 years of age [56]. These ages were excluded for this review. 

With regard to the gender of the participants, three studies (12%) were conducted only in males [44,50,59]. A total of 8 out of 23 (32%) reported results for each sex [38,39,40,42,43,49,53,61]. The other studies (56%) did not differentiate the children’s sex in the reported results.

The studies included anthropometric measures by clinicians, such as birth weight and body mass index z score (zBMI), and measures of adiposity such as body fat accumulation or abdominal circumference. Different reference charts and guidelines were used to standardize measures (see Table 1). Additionally, other parameters related to obesity or metabolic syndrome, such as Leptin, IGF, Insulin, or Adiponectin were measured during postnatal ages.

The most common pesticides analyzed were organochlorines (20 out of 25, 80%) DDE, DDT and their metabolites (p,p′-DDE/p,p′-DDD and p,p′-DDT), Hexachlorobenzene (HCB) (11 out of 25, 44%), and β-hexachlorocyclohexane (βHCH) (6 out of 25, 24%). Chlordecone was measured in one study [54]. Other pesticides like Dichlorophenols (DCPs) were measured in one study [60]. Pyrethroids were also measured in one study [38]. Organophosphate pesticides were detected by six nonspecific dialkylphosphate (DAP) metabolites of diethylphosphate (DEP), diethylthiophosphat (DETP), diethyldithiophosphate (DEDTP), dimethylphosphate (DMP), dimethylthiophosphate (DMTP), and dimethyldithiophosphate (DMDTP) and the sum of these compounds (diethylphosphate metabolites) (DE; sum of DEP, DETP, and DEDTP), dimethylphosphate metabolites (DM; sum of DMP, DMTP, and DMDTP), and dialkylphosphate metabolites (DAP; sum of DM and DE) [53].

Prenatal exposure to pesticides was assessed by the determination of biomarkers in the mothers during pregnancy in blood [40,41,42,47,49,51,52,56], urine [38,53], or both [55]. Some studies measured the exposure to pesticides at delivery in cord blood samples [37,43,45,46,48,53,54,61] or maternal blood [44,57]. Postnatal exposure to pesticides was measured in blood [39,47,50,56,59], breast milk [56,58], or urine samples [38,60].

#### 3.2.1. Outcomes and Exposure Statement

##### Organochlorines Pesticides: p,p′-DDE/p,p′-DDD and p,p′-DDT

Prenatal p,p′-DDE/p,p′-DDD and p,p′-DDT levels were found to be significantly associated with an increase in neonatal birth weight in both sexes [52], or in girls [61]. DDE was positively associated with overweight or elevated BMI at 6, 12 or 14 months of age [39,46,51]. Other studies found prenatal DDE exposure to be associated with a reduction of BMI scores at 6 months of age [46] or even no association with DDE prenatal exposure and BMI at 14 months [48].

During childhood or adolescence (4–16 years), positive associations were reported between DDT/DDE, p,p′-DDE prenatal exposure and BMI or overweight risk [37,43,45,47,55,58,62], waist circumference [47], waist circumference/abdominal obesity, and waist/height ratio only in girls [43,48]. Associations between prenatal exposure to DDT and DDE and several measures of obesity (body mass index z score, waist circumference z score) at 9 and 12 years of age were found in boys but not in girls [40,49]. Other studies found no association with p,p′-DDE prenatal exposure and BMI or central adiposity measures [37,41,42,44,57].

By contrast, there was no relationship between p,p′-DDE postnatal exposure and weight gain during childhood or adolescence [56]. However, DDE postnatal exposure showed a significant relationship with other indicators related to obesity, such as leptin concentration in serum of adolescent boys [59]. Finally, boys with higher serum p,p′-DDE were reported to have significantly lower mean zBMI [50].

An association has been demonstrated between pesticide exposure and adiponectin and insulin levels in serum. A decrease in adiponectin levels was associated with an increase in DDE levels. Furthermore, a decrease in insulin levels was associated with an increase in DDE, only in girls [53].

##### Organochlorines Pesticides: HCB, β-HCH, Chlordecone

HCB was found to be significantly associated with an increase in neonatal birth weight, with a special emphasis on girls [61]. HCB was positively associated with overweight or increased BMI at 14 months of age up to 7 years of age [37,39,45,55]. This association is stronger in girls [46]. HCB was also associated with excess adiposity [37,47]. However, higher serum HCB and β-HCH were related to significantly lower mean zBMI [50]. Other authors found that there was no association of HCB with BMI in childhood [41,48,51].

Higher prepubertal HCB concentrations were associated with greater ratios of insulin resistance, higher serum insulin, and the homeostatic model assessment of insulin resistance (HOMA-IR) levels [59]. No relationship of postnatal HCB levels with weight gain was found for any of the ages studied [56].

In the case of β-HCH exposure, a positive association was reported between β-HCH prenatal exposure and BMI and overweight risk at 12 months [46] or 7 years [55], but study [51] found no association. A decrease in insulin levels was also associated with a low concentration of β-HCH in newborns [53]. Moreover, postnatal exposure to β-HCH, did not show effects in obesity related parameters [59].

Finally, the chlordecone exposure was not associated with any change in birth weight [54].

##### Pyrethroids, Diclorophenols (DCPs), Organophosphatades, and Mixed Pesticides

In terms of pyrethroids exposure, differential effects were reported depending on the time of exposure. In this regard, prenatal urinary 3-phenoxybenzoic acid (3-PBA) concentration was not associated with height, weight, or zBMI at 4 years of age, regardless of sex [38]. However, postnatal childhood urinary 3-PBA concentration measured at 4 years of age was positively associated with zBMI in 4-year-old girls [38].

Regarding dichlorophenols, a cross sectional study of postnatal exposure found a positive association between 2,5-DCP zBMI, waist circumference and obesity, and a negative association with total cholesterol and HDL-C was detected at 6–18 ages. 2,4-DCP showed an association with HDL-C [60].

In the case of the organophosphate pesticides, one mixed effect study of various DAP metabolites reported by [53] showed an increase in insulin levels with higher concentrations of DAP metabolites, specific with DM metabolites, which was further reinforced by adjustment for BMIz scores at birth.

In a study that analyzed the role of combined pesticides in relation to birth weight [61], a positive association between the proportion of newborns with small gestational age that have been exposed to ≥3 different OCPs was reported among boys.

### 3.3. Animal Studies

A total of 16 studies that described the relationship between pesticides and obesity were eligible. From these studies, seven were excluded due to the animal model screening used and a total of 9 studies were finally included (Table 2).

All eligible articles were experimental studies and evaluated different pesticides. Seven studies were done with rats (Sprague Dawley, 44.4%; Long Evans, 22.2%: Wistar, 11.1%) and two with mice (C57BL/6J and CD1).

Three publications (33.33%) used male animals and six (66.66%) used mixed populations. Sample size per group ranged from 4 to 10 animals. In particular, seven studies used insecticides: parathion postnatally [63,64,65], dichlorodiphenyltrichloroethane [65], imidacloprid, oxamyl and lambda cyhalothrin [66] prenatally, chlorpyrifos prenatally [66,67] or postnatally [68], and insecticide, miticide endosulfan prenatal and/or postnatally [69]. One of these studies used herbicides at different stages of development: atrazine during pregnancy and lactation [70], and another study used the fungicide vinclozolin during pregnancy and lactation [71]. Six studies used gastroesophageal/gavage administration (66.6%) and three used subcutaneous (33.4%) administration.

All studies measured body weight from lactation to adolescence [64,66,69,70,71] or during adolescence [63,64,65,68,69]. La Merrill et al. (2014) [65] calculated body weight and percent fat mass by MRI. Additional parameters linked to obesity were measured during postnatal age such as body volume, specific gravity, core temperature, and energy expenditure [65], serum leptin levels [67], liver function enzymes, oxidative stress, and lipid peroxidation [66], histomorphological study of the hippocampus and protein expression and mRNA levels in the hippocampus [70].

In addition, four studies reported behavioral tasks. Depressive-like behavior was evaluated using the forced swim test (FST), learned helplessness (LH) test, or novelty-suppressed feeding test (NSFT) [68]. The Morris water maze (MWM) was used to assess spatial learning and memory [70]. Finally, a straight runway was used to evaluate the extinction and acquisition of a conditioned running response [71].

All selected preclinical studies regarding pesticide exposure and obesity are summarized in Table 2. 

#### 3.3.1. Outcomes and Exposure Statement

##### Obesity

There is limited available data on the effects of pesticides on weight gain. A relatively small number of studies reported a link between insecticide exposure and increased body weight gain, while the rest of the investigations found inconsistent results. Only 3 out of the 9 studies were labeled as H quality [65,69,71], 4 were labeled as MH [63,64,66,70], and two others as ML [62,68]. There were no preclinical studies labeled as VL, L or VH quality (summarized in Table 2).

More specifically, one study found an association between organochlorine insecticide exposure (DDE) and weight gain, but only in female offspring [65].

Exposure to organophosphorus insecticides reported conflicting results. Accordingly, body weight seems to be related with chlorpyrifos dosage, time of exposure to pesticide, and sex. In this regard, prenatal and postnatal exposure to chlorpyrifos at very low doses was reported to increase body weight in male rats, but not in female offspring [63]. However, high doses of chlorpyrifos during the prenatal period has not been found to have any relationship with obesity in male and female adult rats [66].

Regarding the effects of postnatal exposure to pesticides, no relationship was found between exposure to chlorpyrifos and the body weight of adult male rats [68]. In addition, Lassiter et al. (2008, 2010) [63,64] found that parathion was positively associated with overweight in male rats and decreased body weight in female rats.

On the other hand, there is limited available data on the effects of pyrethroids, carbamates, and neonicotinoids on weight gain. Only one study did not report any effects of prenatal exposure to lambda cyhalothrin (a pyrethroid), oxamyl (a carbamate), or imidacloprid (a neonicotinoid) on the body weight of adult rats [66].

Lastly, no relationship was found between perinatal exposure to atrazine [70] and prenatal/postnatal exposure to endosulfan sulfate and obesity in young experimental animals [69]. Likewise, there were no effects of vinclozolin on pup body weight [71].

##### Physiological, Biochemical, Metabolic, and Behavioral Measures

Organophosphorus insecticides such as chlorpyrifos caused oxidative stress as well as dysregulation on antioxidant enzymes levels in the liver and kidney of rat offspring at weaning and adulthood [66]. Likewise, prenatal exposure to chlorpyrifos increased malondialdehyde (MDA), alanine transaminase (ALT) and aspartate transaminase (AST) levels in rats. Similar results were seen after prenatal exposure to lambda-cyhalothrin imidacloprid and oxamil [66]. However, the pesticide combination (imidacloprid + lambda cyhalothrin) administered at low dose did not affect liver function enzymes [66].

Furthermore, exposure to atrazine during pregnancy and lactation was associated with impaired long-term spatial orientation using the Morris water maze and morphological damage of CA1 subfield of the hippocampus [70].

On the other hand, improved serum glucose level and fat metabolism were found in male rats, but impaired serum glucose level and lipid metabolism was detected in female rats after low doses of parathion [63]. Likewise, a positive association was found between decreased serum adiponectin levels in male rats and increased tumor necrosis factor alpha (TNFα) in adipose tissue of rats of both sexes.

Finally, a positive correlation has been found between postnatal exposure to chlorpyrifos and depressive-like behavior in adolescent male rats [68].

## 4. Discussion

In this systematic review about the effect of exposure to pesticides in humans we have included large scale longitudinal cohort studies with long follow up periods that allow for model associations including adjustment for covariates. These types of studies are important to understand the clinical impact of exposure to pesticides on human health. Only two studies were cross-sectional and were retained in this systematic review due to the high number of participants or the specificity of the pesticides studied. There is heterogeneity in the population studied with different age groups, diverse lifestyle habits and socioeconomics levels. Some populations are from rural areas in which a high level of exposure to pesticides can occur in mothers and children, and other studies consider all the available population in a given area which may result in differences in the exposure level of study participants, even if from the same country.

Another interesting aspect is the diversity in the outcomes studied. A diagnosis of obesity in children based solely on weight data, without taking into account children’s growth, does not seem like the most appropriate outcome to use. For this reason, most studies have used zBMI as the primary outcome, adjusted to an international or national chart to facilitate the comparison between studies. Some studies have reported measures of BMI at a single point in time. This approach is incomplete and ignores the potential impact of children’s growth [72]. Some authors have proposed that circulating levels of OCP can vary depending on weight gain or loss [73] and the association of OCP and BMI in growing children depends on growth rates, and other adiposity indicators such as amount and type of fat tissue [5,73]. Some studies have included the measure of abdominal adiposity, where children with waist circumference in the ≥ 90th percentile are considered at risk of metabolic syndrome [74], but this outcome is not considered in all studies and can be different depending on the characteristic of the population studied. The difference in the outcomes reported makes it difficult to conduct an appropriate comparison of the studies.

When we examined the effect of prenatal exposure of OCP at birth, we found that increased levels of OCPs (p,p′-DDE/p,p′-DDD and p,p′-DDT, HCB) have a positive association with an increase in neonatal birth weight in girls. This OCP tendency in birth weight is not consistent when children are examined at 14 months of age. DDE was positively associated with overweight at 14 months of age, but another study found no association with DDE prenatal exposure and BMI at 14 months. This inconsistence could be explained due to the normal growth of children. It seems that birth weight could not be an appropriate predictor for future obesity. After a peak in the first year of life, BMI declines between age 4 and 6 [75]. For this reason, some authors have proposed the use or rapid growth scores [51], a measure obtained between birth and 6 months standardized to World Health Organization data.

During childhood (4–12 years), positive associations were reported between DDT/DDE, p,p′-DDE prenatal exposure and BMI, overweight risk or in adiposity measures. Results seem to be affected by sex as markers of abdominal fat were found to be positively associated with OCPs exposure only in girls aged 7–9 In other studies, associations between prenatal exposure to DDT, DDE, and several measures of obesity (body mass index z score, waist circumference z score) at the ages of 9–12 were found in boys but not in girls. It seems clear that prenatal exposure to DDT and its metabolites have differential effects on each sex. It has been suggested that DDT, DDE and their subproducts have antiestrogenic or antiandrogenic effects. For example, p,p′-DDE and p,p′-DDT have an antiandrogenic effect whereas o,p′-DDT has an estrogenic effect and has been associated with adipose dysfunction [76,77]. In fact, some of these compounds act like endocrine disruptors, and act mainly by interfering with hormones binding to estrogen or androgen receptors, and mimic the natural hormone’s actions [11] and could act differentially in boys and girls but the results are not consistent.

Other studies found no association with p,p′-DDE prenatal exposure and BMI. These results may be due to the fact that in these studies, subjects were exposed to high prenatal levels of OCP. However, prenatal exposure to DDT was associated with overweight or high BMI in studies that included populations with low levels of exposure. Some studies reported an increase in zBMI with increased prenatal exposure to other organochlorine compounds such as HCB and β-HCH, as well as increased overweight rate at the age of 7. HCB was also associated with excess adiposity in childhood. By contrast, there was no association between HCB and zBMI during childhood in other studies. Other parameters associated with obesity, such as insulin levels at birth, showed that decreased insulin levels were associated with low concentrations of β-HCH in newborns. However, organochlorides pesticides have been described to have an effect at low doses, similar to low dose effect of endocrine disruptors [78]. Besides that, it is necessary to consider that there are differences in the levels of exposure of each cohort used. For example, studies using CCP cohorts have higher pesticide levels than other US cohorts and the number of people suffering obesity or overweight is lower than in the US general population.

It is important to note that postnatal values of pesticide exposure are not as clearly associated with obesity or overweight. A study reported that boys with higher serum HCB and p,p′-DDE had significantly lower mean BMI z-scores [50] but another study found no relationship with weight gain in boys or girls [58]. However, there is some evidence that an effect on hormonal status and HCB pesticides exists, given that higher prepubertal HCB concentrations were associated with greater ratios of insulin resistance, higher serum insulin, and HOMA-IR levels [59].

Other compounds such as pyrethroids showed that prenatal exposure had not shown any effects, but childhood urinary 3-PBA concentration measured at 4 years of age was positively associated with BMI z-scores in 4-year-old girls [38].

Only one study reviewed here investigated the association of postnatal DCP presence and obesity related outcomes in a population with similar exposure levels [60]. The association between 2,5-DCP and increased zBMI or obesity seems to be consistent, and it is found in all studies. With regard to the other compounds studied, results are inconsistent, showing a positive association with overweight but not with obesity. However, this study is cross sectional with data collected at a specific point in time hence causation cannot be assumed. DCPs are rapidly metabolized and excreted so a single measurement may not show the effect of long-term exposure. Moreover, DCP can be stored in fat tissues, so people can have a different exposure depending on their own adipose tissue.

In the case of the organophosphate pesticides, one mixed effect study of various DAP metabolites showed an increase in insulin levels with higher concentrations of DAP metabolites [53]. This is in line with other authors reporting that OCP early exposure causes hyperglycemia and hyperinsulinemia [79,80].

One concern in the human exposure to pesticides is the fact that it is difficult to disentangle the individual effect of a single pesticide given that all the studied population present more than one pesticide in the organism. In this context, it is difficult to understand if the effect found is due to a single compound or to a combined effect of pesticides. Moreover, throughout life, people are exposed to pesticides from different sources every day, [81] and it is possible that the harmful effect of pesticides on health may also be due to an accumulative or chronic effect. For example, OCP have shown great persistence in the environment, bioaccumulation in the food chain, and storage in the human adipose tissues [82]. In fact, OCPs have been detected in high amounts in the serum of adults aged 60 and older in USA (for revision see [82]). Studies simultaneously considering several exposures, or other environmental factors are needed [83,84].

The human evidence is diverse, and difficult to compare due to the methodological differences between studies and the diverse pesticides evaluated. It seems that exposure to pesticides has an effect on some parameters related with obesity or adiposity factors. However, the underlying mechanisms or processes remain unclear.

For some years now, both preclinical and clinical endocrinological studies have revealed that hormone-disrupting chemicals can have detrimental effects on many health problems [18,85]. These conclusions are based on observational human epidemiological studies and experimental animal studies. Therefore, research in animal models is essential to improve our understanding about the effects of early exposure to pesticides in our health. Animal experimentation is essential and required before starting with clinical studies, aimed to prevent, and to close knowledge gaps regarding the causes and pathophysiology of human disease.

The systematic review of the effects of pesticides on obesity in experimental animals showed inconsistent results, mainly due to the small number of studies found, the wide range of drug dosage and administration routes, the different species and strains of rodents used, and the heterogeneity of research objectives.

In this regard, the study that analyzed the effects of exposure to organochlorine compounds on obesity reported a significant association in rodents. In particular, perinatal DDT exposure showed sexually dimorphic effects in mice. Perinatal DDT exposure caused an increase in body weight and adiposity of young adult female mice associated with impaired thermogenesis and energy expenditure. In addition, perinatal DDT exposure combined with HFD caused dyslipidemia in females, but not in male mice. Accordingly, DDT is regarded as an endocrine-disrupting (ED) chemical that can alter hormone-dependent functions, including several behaviors modulated by neuroendocrine systems [86]. One of the suggested mechanisms of action of DDT is through its estrogenic and androgenic effects on target tissues [87], particularly affecting the thyroid gland.

Endocrine activity of DDT has the potential to cause numerous adverse outcomes, including the disruption of several endogenous physiological processes [88]. In this way, low perinatal DDT doses have been associated with more pronounced hypothyroidism in female rats after exposure to DDT [89]. Hypothyroidism is correlated with decreased thermogenesis, decreased metabolic rate, and it has also been shown to correlate with a higher BMI and a higher prevalence of obesity in experimental animals and humans [90,91,92]. Furthermore, organochlorine pesticides are substances designed to be very resistant to chemical degradation, and are therefore still present in the food chain, persisting with detectable levels during decades in human tissues [93], and may induce epigenetic transgenerational inheritance [94,95].

Like organochlorine exposure, there is no clear scientific evidence to support a relationship between prenatal exposure to pyrethroids, carbamates, or neonicotinoids and overweight in experimental animals. In this regard, the pyrethroid lambda cyhalothrin is associated with reproductive toxicity and degenerative damage in testes, liver, kidneys, and spleen [96] and it can cause oxidative damage to the kidney and brain of rodents [97,98]. Similarly, although imidacloprid promoted adipogenesis and insulin resistance [27], it was not directly associated with obesity [99,100].

Moreover, we found one study that assessed the effects of prenatal/lactational exposure to glyphosate herbicides on obesity. However, no relationship was reported between herbicide exposure and obesity. In fact, previous studies found similar results in rat and mice [101,102]. In this regard, the administration timing of herbicides is critical. Administration of atrazine during sexual development at low doses has been associated with increased total and cumulative weight gain [103] as well as with decreased body, liver, and testis weight [104] in male mice. However, increased body weight and metabolic function in male mice has been reported previously [103].

In our review, the prenatal exposure to vinclozolin, an antiandrogenic fungicide, has not been related to obesity. In this regard, it should be noted that in utero exposure to vinclozolin is linked to multigenerational phenotypic and epigenetic effects. In particular, vinclozolin exposure has been associated with increased obesity rate in F3 generation female rats [105] and increased body weight in F2 generation male rats [106].

The animal studies reviewed here collectively support the hypothesis that exposure to herbicides induces neurotoxic effects. Thus, there is strong scientific evidence on behavioral and emotional disorders associated with biochemical alterations in the brain after prenatal exposure not only to glyphosate herbicides but also to vinclozolin [107,108].

The organophosphorus insecticide chlorpyrifos seems to be one of the potential obesogenic worldwide [109,110]. However, the results of our analysis seem to be contradictory. In this way, only low doses of prenatal chlorpyrifos exposure led to sex-specific body weight gain in male rats. However, prenatal exposure to high doses or postnatal exposure to chlorpyrifos did not cause overweight in rodents. Some studies have suggested that the main mechanisms of action of chlorpyrifos toxicity are related to oxidative damage, fatty-acid synthesis, and lipid peroxidation [111]. These mechanisms may lead to metabolic disruption, such as insulin resistance and changes in body weight, as observed in adult mice exposed to CPF [112]. In this regard, chlorpyrifos seems to have an influence over the leptin and insulin signaling pathway [113] and on the adipogenic process facilitating lipid storage [110]. Some studies have shown that the gut microbiota could play an essential role in these effects [114,115]. In the same way, chlorpyrifos could have significant effects on endocrine regulation and modulate the development of neuroendocrine pathways and sexual differentiation [111]. In fact, several studies supported the hypothesis that low-dose chlorpyrifos acts as a developmental neurotoxicant [116,117,118]. This would explain the sexually dimorphic effects of gestational exposure to chlorpyrifos on the risk of neurobehavioral disorders in children and experimental animals [117,118,119,120].

Finally, studies about the effects of postnatal exposure to parathion in relation to body weight were found. These studies showed that parathion exposure was sexually dimorphic and dose-dependent in terms of body weight. In fact, sex differences in the effects of parathion in rats have been attributed to the greater susceptibility of females due to the activity of sexual hormones [121]. However, to our knowledge there are no additional studies regarding the effects of parathion in both sexes.

Our study has some limitations. The differences in the design of the studies, (for example in the type and its measurement of exposure), in the detection of OCPs in the samples, and the variety of outcomes reported, prevent to draw any definite conclusions. Another limitation is the narrow range of exposure to OCPs across human populations that may not reflect the broad dose–response relationship. Finally, it should be noted that the studied populations would have been chronically exposed to a complex mixture of OCPs and other pollutants, instead of a single chemical exposure as reported in the selected studies. The search strategy was designed to identify multiple outcomes from multiple steams of evidence within a specific time frame. More specific search strategies, for example based on specific outcomes, and including increasingly aged population may provide a general overview of the effects of OCP exposure on health. Another limitation is that the sexually dimorphic nature of obesity is not adequately addressed in all studies. If possible, future studies should also incorporate female animals to evaluate sex differences after pesticide exposure in preclinical models.

## 5. Conclusions

This systematic review reveals that there is still scarce evidence to support a clear relationship between exposure to pesticides and obesity in humans and experimental animals. It seems clear that effects of OCP on body weight and metabolic functions depending upon type and dose of the chemical, the timing of exposure, and the metabolic route. The effects of pesticide exposure on body weight change are mostly inconclusive and report conflicting results. These outcomes are dependent on many factors, including dosage and route of administration, species, sex, and treatment duration. In humans, a long-term life exposure to mixed pesticides makes it necessary for more studies to disclose the impact of the combined effects of different pesticides on human health.

More research is needed to improve understanding of whether repeated exposures over time or just short-term exposures to pesticides during critical windows of development are related to obesity. Finally, this area of research could benefit from the application of exposomic methods, that can yield more integrated views about combined effects of multiple exposures to a particular phenotype.

## Figures and Tables

**Table 1:** Effects of pesticide exposure on risk of obesity in clinical studies.

Study, Year (Reference)	Study Design Region	Age at Evaluation/Sex/Sample Size/Rural vs. Urban	Type/Agent/Source of Exposure Assessment	Physiological Assessment in Children	Physiological Outcomes	Quality Index
Güil-Oumrait et al. [37]	INMA Cohort/Menorca (Spain)	4, 6, 11, 14, 18 yo/ Both/N = 379/Rural	Env/p,p′-DDT, p,p′-DDE, HCB, PCBs/Cord blood	Birth weight, WC, HDL-C, LDL-C, triglycerides, insulin, and glucose.	Prenatal p,p′-DDT and HCB concentrations were significantly associated with increased BMI during childhood and adolescence (from 4- to 18-years-old), as well as WHtR during adolescence. Positive association between prenatal HCB and body fat % in adolescence. A continuous increase in HCB was associated with an elevated body fat % across all ages, and with higher CM-risk score and lipid biomarkers (total cholesterol, triglycerides and LDL-C at 14 years). p,p′-DDT exposure was associated with an increased CM-risk score, and ΣPCBs concentrations with LDL-C in all adolescents, and with total cholesterol only in girls.	8 VH
Lee et al. [38]	CAS Cohort/Seoul and Gyeonggi (Korea)	4 yo/ Both/N = 578/Urban	Env/Pyrethroid/Maternal urine (14–27 gw) and postnatal (4 yo) urine samples	zBMI	Prenatal urinary 3-phenoxybenzoic acid (3-PBA) concentration was not associated with height, weight, or zBMI at 4 years of age, regardless of sex. Postnatal childhood urinary 3-PBA concentration measured at 4 years of age was positively associated with zBMI in 4-year-old girls	8 VH
Warner et al. [49]	CHAMACOS Cohort/California (USA)	12 yo/ Both/N = 240/Rural	Env/DDT, DDE/ Gestational maternal blood (26 gw) or delivery blood samples	zBMI and zWC	Associations between prenatal exposure to DDT and DDE and several measures of obesity (zBMI, zWC) at 12 years of age in boys but not in girls	8 VH
Xu et al. [52]	Cohort/Shengsi Islands (China)	Birth/ Both/N = 106/Rural	Env/DDT/ Cord blood (delivery) samples	Birth weight	Prenatal DDT levels were found to be significantly associated with an increase in neonatal birth weight in both sexes	8 VH
Debost-Legrand et al. [53]	PELAGIE Cohort/Brittany (France)	Birth/ Both/N = 268/Rural	Env/DDE, βHCH, DAP/ Prenatal urine samples (1st trimester) Cord blood (delivery) samples	Insulin and adiponectin levels	A decrease in adiponectin levels was associated with an increase in DDE levels. Decrease in insulin levels was associated with an increase in DDE only in girls. A decrease in insulin levels was associated with low concentration of β-HCH in newborns. An increase in insulin levels with higher concentrations of DAP metabolites, specific with DM metabolites, reinforced by adjustment for BMIz scores at birth	8 VH
Hervé et al. [54]	TIMOUN Cohort/Guadeloupe (FWI)	Birth/ Both/N = 593/Rural	Env/Chlordecone/ Cord blood (delivery) samples	Birth weight	No association between prenatal chlordecone exposure with birth weight	8 VH
Agay-Shay et al. [55]	INMA Cohort/ Sabadell (Spain)	7 yo/ Both/N = 470/ Urban	Env/DDE, HCB, βHCH/ Prenatal urine samples (1st and 3rd trimester) Maternal blood (1st trimester)	Overweight and zBMI	Positive associations between DDE and zBMI or overweight risk. An increase in zBMI and overweight was found with prenatal HCB exposure.	8-VH
Tang-Peronad et al. [56]	Cohort/Odense (Denmark)	8–10, 14–16, 20–22 yo/ Both/N = 278/Rural	Env/DDE, HCB/ Postnatal blood samples and breast milk	zBMI, WC and SFT	No relationship of postnatal HCB levels with weight gain was found in any age studied	8 VH
Høyer et al. [57]	INUENDO Cohort/Warsaw (Poland)	5–9 yo/ Both/N = 1109/Rural	Env/p,p′-DDE/ Gestational maternal blood (2nd–3rd trimester)	zBMI	No association with p,p′-DDE prenatal exposure and BMI	8 VH
Tang-Peronad et al. [58]	Cohort/Faroe Islands (Denmark)	5, 7,5 yo/ Both/N = 539/Rural	Env/DDE/ Gestational maternal blood (34 gw) and breast milk samples	BMI and WC	Positive association was reported among DDE prenatal exposure and BMI	8 VH
Valvi et al. [39]	INMA Cohort/Sabadell, Valencia and Gipuzcoa (Spain)	6, 14 mo/ Both/N = 136/ Urban	Env/DDT, DDE, HCB/ Gestational maternal blood (7th–26th gw) samples	zBMI	DDE and HCB was positively associated with overweight at 14 months of age. HCB was positively associated with overweight at 14 months of age	8 VH
Warner et al. [40]	CHAMACOS Cohort/California (USA)	9 yo/ Both/N = 261/Rural	Env/DDT, DDE/ Gestational maternal blood (26 gw) or delivery blood samples	zBMI and zWC	Associations between prenatal exposure to DDT and DDE and several measures of obesity (zBMI, zWC) at 9 years of age in boys but not in girls	8 VH
Cupul-Uicab et al. [41]	Cohort/CPP (USA)	7 yo/ Both/N = 1915/Both	Env/HCB, βHCH, p,p′-DDE, p,p′-DDT/ Gestational maternal blood (3rd trimester) samples	zBMI	No association with p,p′-DDE prenatal exposure and BMI. No association of HCB with zBMI in childhood	8-VH
Warner et al. [42]	CHAMACOS Cohort/California (USA)	7 yo/ Both/N = 270/Rural	Env/DDT, DDE/ Gestational maternal blood (26 gw) samples	zBMI	No association with DDE prenatal exposure and zBMI	8 VH
Valvi et al. [43]	Asthma Multicenter Infants Cohort/Menorca (Spain)	4, 6,5 yo/ Both/N = 344/Rural	Env/DDT, DDE, HCB/ Cord blood (delivery), postnatal blood (4 yo) samples	zBMI	Positive associations were reported between DDT/DDE prenatal exposure and BMI	8 VH
Cupul-Uicab et al. [44]	Cohort/Chiapas (Mexico)	13, 30 mean mo/ Males/N = 789/ Both	Env/DDE, DDT/ Maternal blood (delivery) samples	Heigh, weight SDS and BMI	No association with DDE prenatal exposure and BMI at 14 months	8-VH
Smink et al. [45]	Asthma Multicenter Infants Cohort/Menorca (Spain)	6,5 yo/ Both/N = 405/Rural	Env/HCB/ Cord blood (delivery) samples	zBMI	Increase in z BMI and overweight at age 5–7 was found with prenatal HCB exposure	8 VH
Yang et al. [46]	Cohort/Wuhan (China)	Birth, 6, 12, 24 mo/N = 1039/Urban	Env/αHCH, βHCH, γHCH, p,p′-DDT, p,p′-DDD, p,p′-DDE/Cord blood	zBMI	Higher cord serum βHCH concentrations were associated with higher zBMI at 12 and 24 mo. Higher cord serum γHCH and p,p′-DDT were associated with higher zBMI at 6 and 12 mo. Cord serum βHCH was positively associated with the risk of overweight at 12 mo. Among girls, the effects of βHCH on zBMI and overweight were stronger than boys at 12 and 24 mo.	7 VH
Vafeiadi et al. [47]	Rhea Cohort/Crete (Greece)	4 yo/ Both/N = 689/Both	Env/DDE, HCB/ Gestational maternal blood (3rd–4th gw) postnatal blood samples	MBI, WC, SFT, leptin and adiponectin	Positive associations were reported among DDE prenatal exposure and BMI/WC. HCB was associated with excess adiposity	7 VH
Delvaux et al. [48]	FLEHS Cohort/Flanders (Belgium)	7 to 9 yo/ Both/N = 114/Both	Env/DDE, HCB/ Cord blood (delivery) samples	WC/abdominal obesity and zBMI	Positive associations were reported among DDE prenatal exposure and waist circumference/abdominal obesity, and waist/height ratio in only girls. No association of HCB with BMI in childhood	7 VH
Burns et al. [50]	Russian Childrens’s study Cohort/ Chapaevsk (Russia)	Annually from 8–9 to 12–13 yo/ Males/N = 350/ Urban	Env/HCB, βHCH, p,p′-DDE/ Postnatal (8–9 yo) blood samples	zBMI	Boys with higher serum HCB, βHCH and p,p′-DDE had significantly lower mean zBMI	7-VH
Mendez et al. [51]	INMA Cohort/Sabadell, (Spain)	Birth, 14 mo/N = 518/Urban	Env/DDE, HCB, βHCH, PCBs./Maternal blood (1st trimester)	zBMI	DDE exposure above the first quartile was associated with a doubling of the risk of rapid growth among children of normal-weight, but not overweight, mothers. DDE was associated with elevated BMI at 14 mo.	7 VH
Burns et al. [59]	Russian Childrens’s study Cohort/ Chapaevsk (Russia)	Annually from 8–9 to 12–13 yo Males/N = 318/ Urban	Env/HCB, βHCH and p,p′-DDE/ Postnatal (biennially from 8–9 to 12–13 yo) blood samples	zBMI, Leptin and Homeostatic model assessment insuline resistence (HOMA-IR)	DDE postnatal exposure shows a significant relationship with other indicators related to obesity such as leptin serum. Higher prepubertal HCB concentrations were associated with greater ratios of insulin resistance, higher serum insulin, and homeostatic model assessment insulin resistance (HOMA-IR) levels. Postnatal exposure to β-HCH did not have an effect on obesity related parameters	6-H
Parastar et al. [60]	Cross sectional study/Isfahan (Iran)	Between 6 and 18 yo/Both/N = 242/Urban	Env/CPs Postnatal urine samples	zBMI, WC, TC, LDL-C and HDL-C	Positive association between postnatal exposure to 2,5-DCP and zBMI, WC and obesity. Negative association with TC and HDL-C were detected at ages 6–18. 2,4-DCP showed an association with HDL-C.	7 VH
Cabrera-Rodriguez et al. [61]	Cross sectional study/Canary Island (Spain)	Birth Both/N = 447/ Rural	Env/20 OCPs/ Cord blood (delivery) samples	Birth weight	Prenatal p,p′-DDE/p,p′-DDD and p,p′-DDT levels were found to be significantly associated with an increase in neonatal birth weight in girls. HCB was found to be significantly associated with an increase in neonatal birth weight, with a special emphasis on girls. Positive association between the proportion of newborns with small gestational age that have been exposed to ≥ 3 different OCPs among boys	6-H

Abbreviations: gw—gestational week; gm—gestational month; mo—month old; yo; year old; oc—occupational; env—environmental; b—both; Mn—manganese fungicides; DMTP—dimethylthiophosphate; DEP—diethylphosphate; DDE—dichlorodiphenyldichloroethylene; DDT—dichlorodiphenyltrichloroethylene HCB—Hexachlorobenzene; βHCH- β-hexachlorocyclohexane; Skinfold thickness (SFT) Dichlorophenols (DCPs) low density lipoprotein-cholesterol (LDL-C), high density lipoprotein-cholesterol (HDL-C) Total cholesterol (TC) Chlorophenols (CPs); WC—waist circumference.

**Table 2:** Effects of pesticides exposure on risk of obesity in mice and rats.

Study, Year (Reference)	Strain/Age at Evaluation/Sex	Exposure Agent/Dosage/Route/Duration of Exposure	Rimary Outcome: Body Weight Mesasures	Behavioral/Biochemical/ Physiological Outcomes	Quality Index
La Merrill et al. [65]	Mice (C57BL/6J)/PND5, PND21- 6 postnatal months (BW)/Both	Dichlorodiphenyltrichloroethane 1.7 mg/kg/d Gavage GD11.5-PND5	↓ decreased body weight in males (PND5)	↓ body core temperature, ↑ energy expenditure in females = body core temperature in males	15 H
Yan et al. [69]	CD-1 mice/PND1–15th postnatal week (BW), 15th postnatal week (BM, SS,OT)/M	Endosulfan sulfate 0.03 mg/kg Gavage GDO-PND21	= body weight (PND1–42)		14 H
André et al. [71]	Rats (Long Evans)/PND1–20 (BW), PND60–80 (BT)/Both	Vinclozolin 0.1, 3, 6 or 12 mg/kg/d Gavage GD14-PND3 Vz was not administered on PND0	= pup body weight	Disrupts extinction but not acquisition of a conditioned response in male rats. Male rats were more affected than female rats	14 H
Lassiter et al. [63]	Rats (Sprague Dawley)/PND1–4, PND21–154 (BW,SS)/Both	Parathion 0.1 or 0.2 mg/kg/d s.c. PND1–4	= body weight during PND1–4 ↑ body weight in low dose male group ↓ body weight in low female groups		12 MH
Ndonwi et al. [66]	Rats (Wistar)/PND0–71 (BW, SS)/Both	Imidacloprid 44 mg/kg/d, chlorpyrifos 13.5 mg/kg/d, imidacloprid + lambda cyhalothrin 5.6 + 5.6 mg/kg/d oxamyl 0.4 mg/kg/d Gavage GD0–21	= body weight	↑ aspartate transaminase and alanine transaminase (liver function enzymes), ↑ liver and kidney antioxidants and MDA levels in all the groups Changes in oxidative stress and lipid peroxidation in all the groups	12 MH
Wang et al. [70]	Rats (Sprague Dawley)/pregnancy, lactation, offspring (BW)/1.5–3 postnatal months (BM, BT, SS, OT)/M	Atrazine 100 mg/kg/d Gavage Twice a week GD5—PND21	= body weight (pregnancy, lactation, offspring)	Impaired spatial learning and memory in MWM Histomorphology alterations of hippocampal CA1 area ↓ gene levels of Wnt5a, JNK, PSD95, NR2B, PI3K, and c-fos mRNA in the hippocampus ↓ protein expression levels of Wnt5a, JNK, p-JNK, PSD95, NR2B, PI3 K, and c-fos in the hippocampus 28 days of exercise swimming trainning ameliorated the adverse effects of ATR	12 MH
Lassiter et al. [64]	Rats (Sprague Dawley)/PND21–154 (BW), 22nd postnatal week (SS), 24th postnatal week (BM)/Both	Parathion 0.1 or 0.2 mg/kg/d s.c. PND1–4	= body weight during PND1–4 ↑ body weight in low dose male group		10 MH
Lassiter and Brimijoin [67]	Rats (Long-evans)/PND 21–95 (BW, SS/Both	Chlorpyrifos 1, 2.5, or 4 mg/kg/d Gavage GD7-PND21	↑ weight gain in males beginning at PND51 ↑ body volume in males ↓ specific gravity in males	No effect on brain weight or RNA levels in pups = Serum leptin levels	7 ML
Chen et al. [68]	Rats (Sprague Dawley)/PND37–38 (FST), PND43 (OF), PND46 (NSFT), PND48–52 (LH)/M	Chlorpyrifos 2.5, 5, 10 or 20 mg/kg/d s.c. PND 27–36	= body weight	- FST: ↑ immobility time in the 10 mg/kg dose group - NSFT: ↑ feediLg latency at lower doses (2.5 and 5 mg/kg), ↓ latency at higher doses (10 and 20 mg/kg) - OF: No significant effects on locomotor activity and exploratory behavior measured, respectively by no. of crossings and rearings - LH: ↑ number of escape failures in the 20 mg/kg group	7ML

Abbreviations: BM: Biochemical measures; BW—Body weight; BT: body temperature; BT: behavioural task; CT: core temperature; FST—Force swimming test; LH—Learned helplessness; MDA, Malondialdehyde; MWM—Morris Water Maze; NSFT—Novelty-suppressed feeding test; OM: Obesity Measures; OT: Other tissues; Serum samples: SS; species; WC—waist circumference; s.c., subcutaneous injection; d, day(s); h, hour(s); m, month(s); wk, week(s); PND, postnatal days; GND, gestational day; F—female; M, male; ↑, increase; ↓, decrease; =, no change.

## Data Availability

Not applicable.
